# A microfluidic thermometer: Precise temperature measurements in microliter- and nanoliter-scale volumes

**DOI:** 10.1371/journal.pone.0189430

**Published:** 2017-12-28

**Authors:** Brittney A. McKenzie, William H. Grover

**Affiliations:** Department of Bioengineering, Bourns College of Engineering, University of California Riverside, Riverside, CA 92521, United States of America; Tsinghua University, CHINA

## Abstract

Measuring the temperature of a sample is a fundamental need in many biological and chemical processes. When the volume of the sample is on the microliter or nanoliter scale (*e.g*., cells, microorganisms, precious samples, or samples in microfluidic devices), accurate measurement of the sample temperature becomes challenging. In this work, we demonstrate a technique for accurately determining the temperature of microliter volumes using a simple 3D-printed microfluidic chip. We accomplish this by first filling “microfluidic thermometer” channels on the chip with substances with precisely known freezing/melting points. We then use a thermoelectric cooler to create a stable and linear temperature gradient along these channels within a measurement region on the chip. A custom software tool (available as online Supporting Information) is then used to find the locations of solid-liquid interfaces in the thermometer channels; these locations have known temperatures equal to the freezing/melting points of the substances in the channels. The software then uses the locations of these interfaces to calculate the temperature at any desired point within the measurement region. Using this approach, the temperature of any microliter-scale on-chip sample can be measured with an uncertainty of about a quarter of a degree Celsius. As a proof-of-concept, we use this technique to measure the unknown freezing point of a 50 microliter volume of solution and demonstrate its feasibility on a 400 nanoliter sample. Additionally, this technique can be used to measure the temperature of any on-chip sample, not just near-zero-Celsius freezing points. We demonstrate this by using an oil that solidifies near room temperature (coconut oil) in a microfluidic thermometer to measure on-chip temperatures well above zero Celsius. By providing a low-cost and simple way to accurately measure temperatures in small volumes, this technique should find applications in both research and educational laboratories.

## Introduction

The ability to accurately measure temperatures is a crucial need in many biological and chemical processes [1–4]. For milliliter-scale volumes, conventional thermometers and sensors like thermocouples and thermistors are adequate for measuring the temperature of a substance. However, these techniques are less suitable for measuring the temperature of microliter- or nanoliter-scale volumes (which are commonly encountered with cells, microorganisms, precious samples, and samples inside microfluidic chips). Infrared (IR) thermometers can measure the temperature of a surface [5–7], but their sensitivity to a material’s emissivity and large sensing area make IR thermometers less suitable for measuring the temperature of the fluid inside a microfluidic chip [8, 9]. Similarly, thermocouples affixed to the surface of a microfluidic chip can measure the surface temperature, but there can be significant temperature differences between the surface of a chip and the fluid inside the chip [10]. Resistance temperature detectors (RTDs) can be fabricated inside microfluidic channels [11–15], but RTDs complicate the chip fabrication process and can be physically or chemically incompatible with on-chip fluids. Finally, temperature-sensitive fluorophores, magnetic nanoparticles, and nanodiamond probes can be added to a fluid to measure its temperature [16–19], but these methods require lasers or magnetic fields to activate the probes and may not be chemically or biologically compatible with all samples. In summary, there is an unmet need for simple, broadly-applicable, and label-free techniques for measuring temperatures in small fluid volumes.

**Fig 1:**
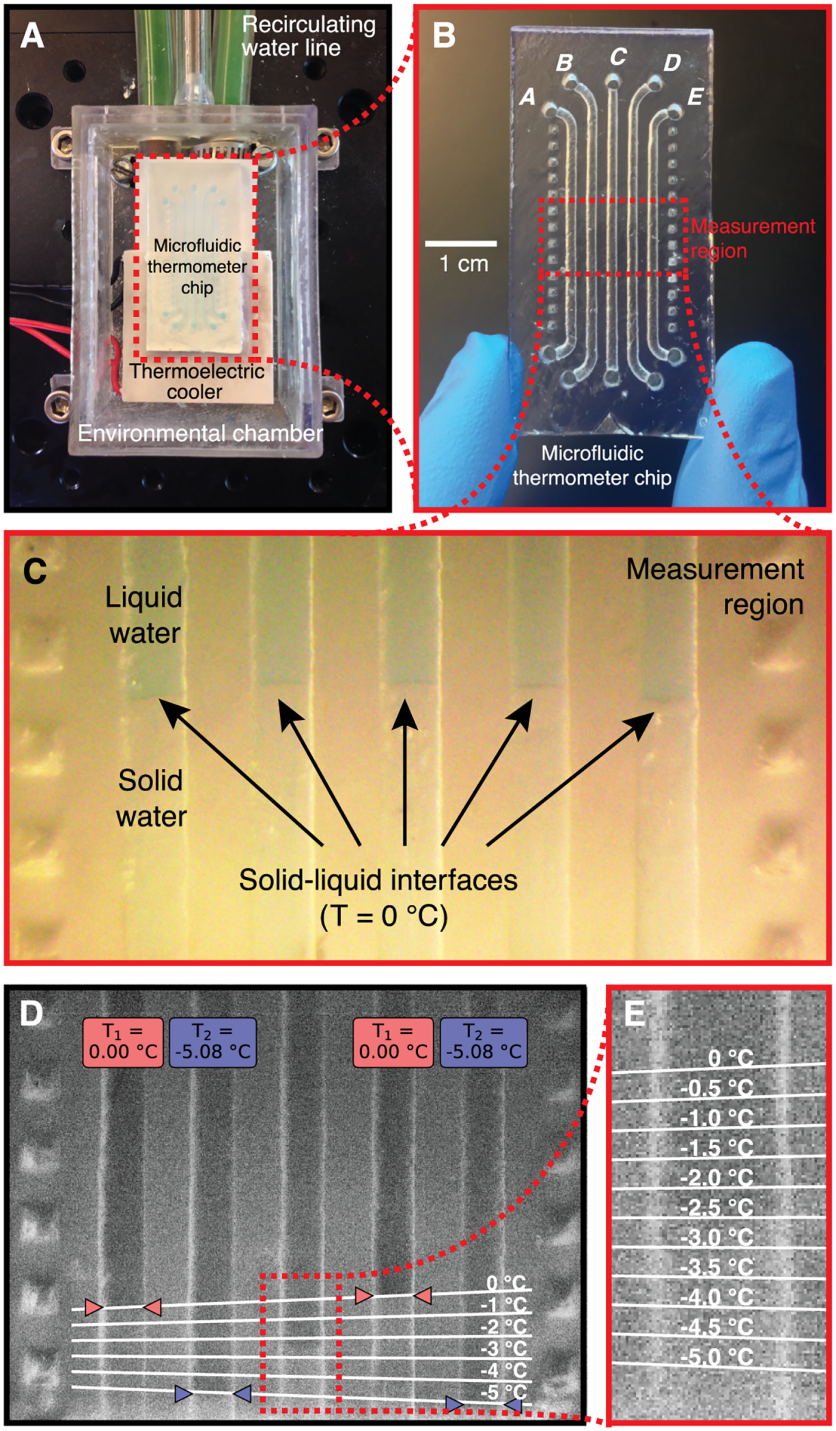
Design and operation of the 3D-printed microfluidic thermometer chip. (A) The chip is placed halfway on a thermoelectric cooler to establish a temperature gradient in the measurement region inside the chip, and the chip is located inside a 3D-printed environmental chamber to eliminate condensation. (B) The thermometer chip includes five channels (*A*–*E*) for containing samples and standards with known freezing/melting points, and a measurement region in which the temperature gradient is roughly linear. (C) In a microscope image of the measurement region while the channels are filled with water, the solid-liquid interfaces are visible and define an isotherm (*T* = 0°C) inside the chip. (D) During use, the user locates solid-liquid interfaces in four channels containing fluids with known freezing/melting points (0°C and -5.08°C in this example), and our software uses these locations to calculate the linear temperature gradient along the channels in this region. (E) Using this gradient, the temperature of the contents of the middle channel can be determined at any point within the measurement region with an uncertainty of about a quarter of a degree Celsius.

In this work we present a “microfluidic thermometer”, a simple microfluidic chip that can measure the temperature of microliter- and nanoliter-scale volumes of fluid with an uncertainty of a quarter of a degree Celsius. The microfluidic thermometer shown in Fig 1 takes advantage of the fact that when two phases of a substance (for example, liquid water and ice) are both present at the same location, the temperature of that location at equilibrium is precisely known (in this example, 0°C). By adding an array of channels to a microfluidic chip, filling those channels with materials with known freezing/melting points, establishing a stable and linear temperature gradient perpendicular to the channels within a measurement region on the chip, and locating the solid-liquid interfaces in the channels, one can visualize the temperature gradient inside the chip and predict the temperature of a sample at any arbitrary point in the measurement region. Solid-liquid interfaces have previously been used for adsorption of proteins [20–22], surfactants [23, 24], and polymers [25] and the formation of lipid bilayers [26], metals and alloys [27, 28], and free radicals [29], but to our knowledge, no previous study has used the locations of multiple solid-liquid interfaces as a tool to measure temperature.

In this proof-of-concept, we created a prototype 3D-printed microfluidic thermometer chip and a custom software tool that can measure the temperature of a 50 microliter sample. We used this chip to measure the “unknown” freezing point of a sodium chloride solution. We also created a 3D heat transfer model of the thermometer chip to visualize the temperature gradient and isotherms inside the device. Finally, we also demonstrated the feasibility of this technique in even smaller (nanoliter-scale) volumes using a glass microfluidic chip. The design of the thermometer chip in standard.STL format (S1 File) and source code for our software tool (S2 and S3 Files) are available for download, meaning that anyone with access to a suitable 3D printer can replicate our technique and use it to analyze their own samples.

## Materials and methods

### Designing and fabricating microfluidic thermometer chips

The microfluidic thermometer chip shown in Fig 1 was designed using SolidWorks (Dassault Systèmes, Vélizy-Villacoublay, France). The chip is 50 mm long, 25 mm wide, and contains five parallel channels with curved entries and exits to provide adequate space for fluid inlets and outlets. Each channel is 1 mm wide, 1 mm deep, and 30 mm long (along the straight portions) with 1.5 mm space between each channel. The chip design was exported as an.STL file (S1 File) and printed using a stereolithography 3D printer (Form 1+, Formlabs, Cambridge, MA) with clear resin (GPCL02; Formlabs). After printing, unpolymerized resin was rinsed away by immersing the chip in isopropanol for 5 minutes, then the device was left to dry overnight.

Additionally, to confirm that our technique can be used with even smaller volumes, a glass microfluidic thermometer chip was fabricated that contains just 400 nL of each fluid. This chip was designed using AutoCAD (Autodesk, San Rafael, CA) and a photomask containing the design was printed using an overhead projector transparency and a conventional inkjet printer. The photomask transparency was placed in contact with a chromium- and photoresist-coated glass photomask blank (Telic, Valencia, CA) and irradiated using ultraviolet light. The exposed regions of photoresist were removed using a developer, and the exposed regions of chromium were removed using a chrome etchant. The now-exposed regions of glass were etched to the desired channel depth using 49% hydrofluoric acid. The remaining photoresist and chromium regions were then removed using acetone and chrome etch, respectively. Fluid inlet/outlet holes were drilled in the glass wafer using diamond-tipped drill bits, and the wafer was bonded to a second (blank) glass wafer using thermal fusion bonding (668°C for 8 hours).

### Preparing liquids with known freezing points

As long as a substance has an identifiable interface between its solid and liquid phases and a precisely-known freezing/melting temperature, the substance could in principle be used in a microfluidic thermometer. In this work we used pure water (freezing point 0°C), sodium chloride (NaCl) solutions with precisely-known freezing points down to −5.08°C [30], and pure coconut oil with a precisely-known solidification temperature of 24.1°C [31]. To enhance the visibility of the solid-liquid interface of the sodium chloride solutions, we added a small amount of blue food coloring to each solution (final concentration 0.01% food coloring by mass). Since coconut oil is transparent when liquid but opaque and white when solid, the solid-liquid interface in coconut oil is easily identified and no food coloring or other additives were needed to visualize this interface. Each channel of the 3D-printed microfluidic thermometer chip received 50 *μ*L of liquid, and each channel of the glass microfluidic thermometer chip received 400 nL of liquid.

### Establishing a temperature gradient across the measurement region on the thermometer chip

A stable temperature gradient was formed across the measurement region on the microfluidic thermometer chip by placing part of the chip on a thermoelectric cooler; the rest of the chip was suspended in air (Fig 1A). The thermoelectric cooler (TEC1-12706, Hebei I.T. Co., Shanghai, China) is connected to a recirculating water line that removes excess heat from the backside of the cooler. To suppress water condensation on the chip (which makes the contents of the chip difficult to visualize), the chip and cooler were placed inside a 3D-printed enclosure that was gently purged with dry nitrogen at 10°C. A glass lid on the enclosure allows for visualization of the thermometer chip. In this manner, we created a stable temperature gradient across the thermometer chip that spans from −20°C (at the end of the chip nearest the cooler) to 10°C (at the suspended end of the chip). For experiments requiring temperature gradients near room temperature (for example, using coconut oil that solidifies at 24.1°C), we simply reversed the polarity of the thermoelectric cooler to make it function as a heater.

### Using the microfluidic thermometer chip

In a typical experiment, the five channels on the thermometer chip (labeled *A*–*E* in Fig 1B) are filled with three different materials. Channels *A* and *D* contain a material with a known freezing point *T*_1_, channels *B* and *E* contain a second material with a known freezing point *T*_2_, and channel *C* contains a material whose temperature is to be measured. While the thermometer chip allows for the measurement of the temperature at any location within channel *C*, in the following analysis we are interested in measuring the temperature at the location where the solid and liquid phases of the material in channel *C* touch—that is, the unknown freezing point *T*_*unk*_ of the material in channel *C*. The chip is then placed on the cooler assembly as shown in Fig 1A and given 20 minutes to reach thermal equilibrium. An inspection microscope (SM-4TZ-144A, AmScope, Irvine, CA) is used to acquire an image of solid-liquid interfaces inside the five channels on the thermometer chip.

The image of the microfluidic thermometer chip is then opened in a custom Python program (S2 and S3 Files). The user specifies the known freezing points *T*_1_ and *T*_2_ of the materials in channels *A*/*D* and *B*/*E*, respectively. The program then instructs the user to click on the locations of the solid-liquid interfaces in all five channels. This provides the program with the (*x*, *y*) coordinates of these interfaces in units of pixels:

- (*x*_*A*_, *y*_*A*_): location of solid-liquid interface in channel *A* at temperature *T*_1_
- (*x*_*D*_, *y*_*D*_): location of solid-liquid interface in channel *D* at temperature *T*_1_
- (*x*_*B*_, *y*_*B*_): location of solid-liquid interface in channel *B* at temperature *T*_2_
- (*x*_*E*_, *y*_*E*_): location of solid-liquid interface in channel *E* at temperature *T*_2_
- (*x*_*C*_, *y*_*C*_): location of solid-liquid interface in channel *C* at temperature *T*_*unk*_

The program then calculates the slope *m*_1_ and y-intercept *b*_1_ of the line between the solid-liquid interfaces of channel *A* and channel *D*:

$$\begin{array}{c} m_{1}=\frac{y_{D}-y_{A}}{x_{D}-x_{A}}\;b_{1}=y_{A}-m_{1}x_{A} \end{array}$$ (1)

and the slope *m*_2_ and y-intercept *b*_2_ of the line between the solid-liquid interfaces of channel *B* and channel *E*:

$$\begin{array}{c} m_{2}=\frac{y_{E}-y_{B}}{x_{E}-x_{B}}\;b_{2}=y_{B}-m_{2}x_{B} \end{array}$$ (2)

These two lines represent isotherms on the thermometer chip; the first line marks a region of the chip at known temperature *T*_1_, and the second line marks a region of the chip at known temperature *T*_2_. The program then calculates where these isotherms intersect channel *C*—in other words, what location (*x*_*C*(*T*_1_)_, *y*_*C*(*T*_1_)_) in channel *C* is at temperature *T*_1_:

$$\begin{array}{c} x_{C(T_{1})}=x_{C}\;y_{C(T_{1})}=m_{1}x_{C(T_{1})}+b_{1} \end{array}$$ (3)

and what location (*x*_*C*(*T*_2_)_, *y*_*C*(*T*_2_)_) in channel *C* is at temperature *T*_2_:

$$\begin{array}{c} x_{C(T_{2})}=x_{C}\;y_{C(T_{2})}=m_{2}x_{C(T_{2})}+b_{2} \end{array}$$ (4)

The program then plots temperature vs. y-coordinate for channel *C* and calculates the slope *m*_3_ and y-intercept *b*_3_ of this line.

$$\begin{array}{c} m_{3}=\frac{T_{2}-T_{1}}{y_{C(T_{2})}-y_{C(T_{1})}}\;b_{3}=T_{1}-m_{3}y_{C(T_{1})} \end{array}$$ (5)

The equation of this line can be used to calculate the temperature of the contents of channel *C* at any point along the channel. By solving this equation using the y-coordinate *y*_*C*_ of the solid-liquid interface in channel *C*, we can determine the unknown freezing point *T*_*unk*_ of the solution in channel *C*:

$$\begin{array}{c} T_{unk}=m_{3}y_{C}+b_{3} \end{array}$$ (6)

The Python code available for download (S2 and S3 Files) automates this process and was used to create Fig 1D and 1E as well as the figures in the *Results and Discussion* section.

### Modeling the microfluidic thermometer chip

To further characterize the shape of the thermal gradient inside the thermometer chip, a 3D model of the chip was created using finite element analysis (COMSOL Multiphysics, Burlington, MA). The model replicates the geometry and temperature of the chip as well as the chip’s orientation partly on the thermoelectric cooler. The “heat transfer physics” module with convective heat flux and a stationary solver was used to model heat transfer between the microfluidic thermometer chip and its channel contents, the cooler, and the surrounding ambient air. Heat transfer coefficients for natural convection normally range from 5 to 50 W m^−2^ K^−1^[32]; however, in the microfluidic thermometer, heat transfer by conduction via the thermoelectric cooler dominates and convective losses are minimal, so we used a slightly lower estimate of the heat transfer coefficient (1 W m^−2^ K^−1^).

## Results and discussion

### Modeling isotherm and thermal isocline shape in the thermometer chip

Our method for analyzing the data from the microfluidic thermometer chip makes two assumptions about the shape of the temperature gradient in the measurement region of the chip:

- First, we assume that if we draw a line between two solid-liquid interfaces that are at the same known temperature in the measurement region on the chip, then all points on that line are also at the same known temperature (the line is an *isotherm*).
- Second, we assume that if we draw a line between two points at different known temperatures within the measurement region on the chip, then there is a linear gradient of temperatures along that line (the line is a *thermal isocline*).

We tested the validity of each of these assumptions using both computer simulations and experimental measurements.

**Fig 2:**
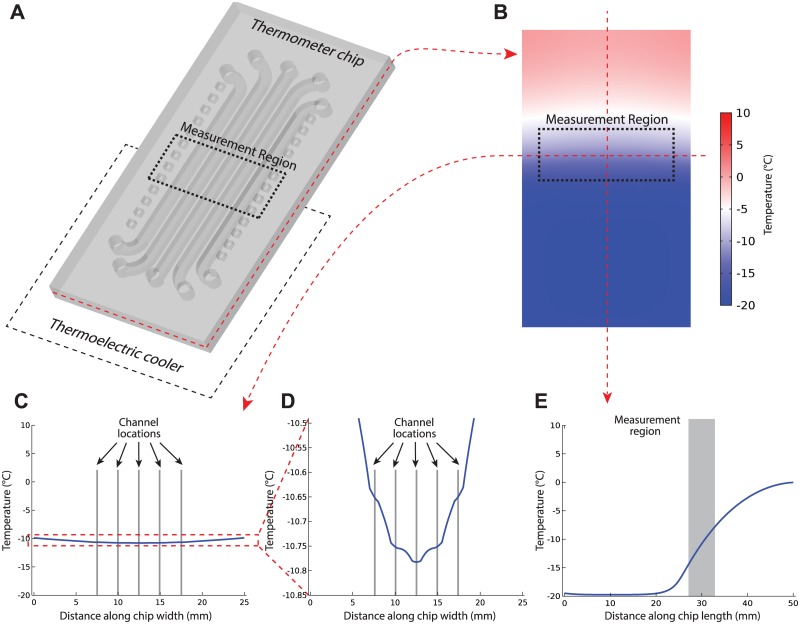
Finite element analysis computer simulations of the 3D-printed microfluidic thermometer chip with all five channels filled with water. (A) The simulated chip is oriented partway on a thermoelectric cooler (*T* = −20°C) and the rest of the chip is suspended in air (*T* = 10°C). By slicing through the middle of the chip, the temperature gradient in the channel plane is visible (B). Plotting the temperature profile in this plane across the five channels in the region where freezing measurements are obtained (C) results in a nearly-flat line in this region; closer inspection (D) shows a variation of only 0.13°C across the five channels, supporting our assumption that isotherms are nearly linear in the measurement region. Plotting the temperature profile in the channel plane along the middle channel (E) results in a fairly complicated temperature profile ranging from a constant −20°C above the cooler to 0°C at the opposite end of the chip. However, in the region of the chip where freezing measurements are observed (corresponding to the shaded region on the plot), the predicted temperature profile is nearly linear; this supports our assumption about the shape of the temperature gradient along the channels in the measurement region.

Fig 2 shows the simulated behavior of the water-filled 3D-printed thermometer chip obtained using COMSOL Multiphysics. In the measurement region on the chip (dotted rectangles in Fig 2A and 2B), the isotherms are straight (Fig 2C) and the temperature across the five channels varies by no more than 0.13°C in the channel region (Fig 2D). These results support our assumption that the isotherms perpendicular to the channels are linear within the measurement region of the chip. Additionally, the temperature gradient is roughly linear along the channel length in the roughly 6-mm-long measurement region (gray area in Fig 2E); this supports our assumption that temperature is a linear function of distance along the channel within the measurement region. Outside of the measurement region on the chip, the temperature profile along the channels is no longer linear but much more complex (see region outside the gray area in Fig 2E). Consequently, solid-liquid interfaces outside the measurement region cannot be used to infer temperatures inside the measurement region.

**Fig 3:**
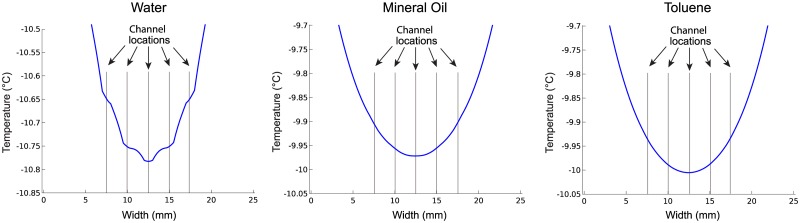
Finite element analysis computer simulations of the temperature profile across the five channels in the measurement region inside the microfluidic thermometer chip, with the channels filled with water (left), mineral oil (center), and toluene (right). Despite the different thermal properties of these fluids (with water being the most thermally conductive), the predicted temperatures differ by less than 0.13°C across the 10 mm wide measurement region. This supports our assumption that isotherms in the measurement region of the thermometer chip are essentially linear, even when filling the channels with materials other than aqueous solutions.

To determine if the contents of the thermometer chip channels affect the thermal behavior of the chip in the measurement region, we repeated the analysis in Fig 2 with the channels filled with water, mineral oil, and toluene. These substances are commonly used in microfluidics and have different thermal properties like thermal conductivity and heat capacity. Fig 3 shows that the different contents of the microfluidic channels had minimal impact on the temperature distribution across the channels in the measurement region.

### Measuring isotherm and thermal isocline shape in the thermometer chip

**Fig 4:**
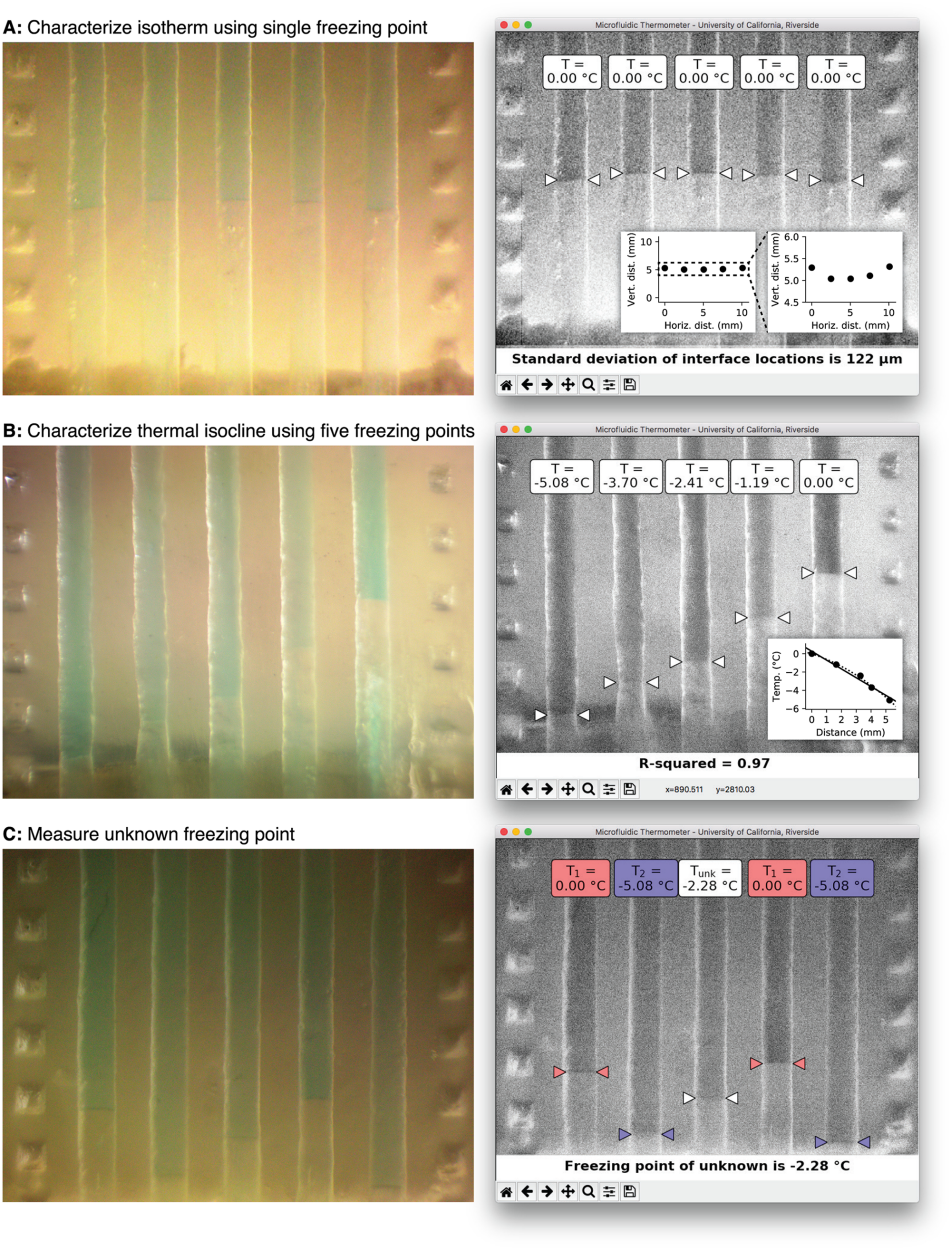
(A) To characterize isotherm shape, all five channels of the microfluidic thermometer chip were filled with water and a stable temperature gradient was formed along the channels using the setup in Fig 1. The solid-liquid interfaces that form are roughly linear. After clicking on the locations of each interface, the software draws an isotherm (0°C) on the image and uses the parameters of this line to estimate the uncertainty of our measurement. The inset shows that the vertical locations of the solid-liquid interfaces differ by less than 500 *μ*m across the entire 10 mm width of the measurement region; this supports our assumption that isotherms are linear in the measurement region. (B) To characterize the thermal isocline shape, each of the five channels were filled with a different solution with precisely-known freezing/melting points (deionized water and 2%, 4%, 6%, and 8% *m/m* NaCl solutions in channels *E*, *D*, *C*, *B*, and *A*, respectively). After clicking on the locations of each interface, the software plots the temperature at each interface vs. the vertical locations of the interfaces. A linear fit (solid line; *R*^2^ = 0.97 and a maximum difference of only 0.48°C between predicted and actual temperatures) confirms our assumption that the temperature gradients are roughly linear in the measurement region. For even higher precision, a second-order polynomial (dotted line) can be used with a maximum difference of only 0.22°C between predicted and actual temperatures). (C) To measure an unknown freezing/melting point, four of the thermometer chip channels were filled with solutions with known freezing points (water in channels *A* and *D*, and 8% (*m/m*) NaCl in channels *B* and *E*) and the remaining channel *C* was filled with a NaCl solution with an “unknown” freezing point. After clicking on the locations of each solid-liquid interface, the software draws isotherms between the known temperatures (0°C at the interfaces in channels *A* and *D*, and −5.08°C at the interfaces in channels *B* and *E*), then calculates the linear temperature gradient between the two isotherms in channel *C* and uses this gradient to determine the temperature at the solid-liquid interface in channel *C* (−2.28 ± 0.26°C). This agrees well with the known literature value for the freezing point of this solution, −2.41°C.

To experimentally verify our assumption that isotherms in the thermometer chip are linear, we filled all five channels in the thermometer chip with the same fluid (deionized water; freezing point = 0°C) and established a temperature gradient along the length of the channels using the setup shown in Fig 1. A micrograph of the solid-liquid interfaces in the measurement region of the thermometer chip is shown in Fig 4A. After importing this image into our custom software (S2 and S3 Files) and clicking on the locations of solid-liquid interfaces in each channel, we found that the solid-liquid interfaces formed a reasonably straight isotherm; the standard deviation of the interface locations in the vertical direction was only 122 *μ*m. Plotting the locations of the interfaces (inset plots in Fig 4A) shows that the vertical locations of the five interfaces differ by less than 500 *μ*m (across all five channels; a horizontal distance of 10 mm). We repeated this experiment for a second solution, an 8% (*m/m*) NaCl solution with a known freezing point of −5.08°C. The resulting interfaces were again visible and linear (standard deviation of vertical interface location = 130 *μ*m; data not shown). These results further support our assumption that isotherms are linear in the measurement region of the chip.

**Fig 5:**
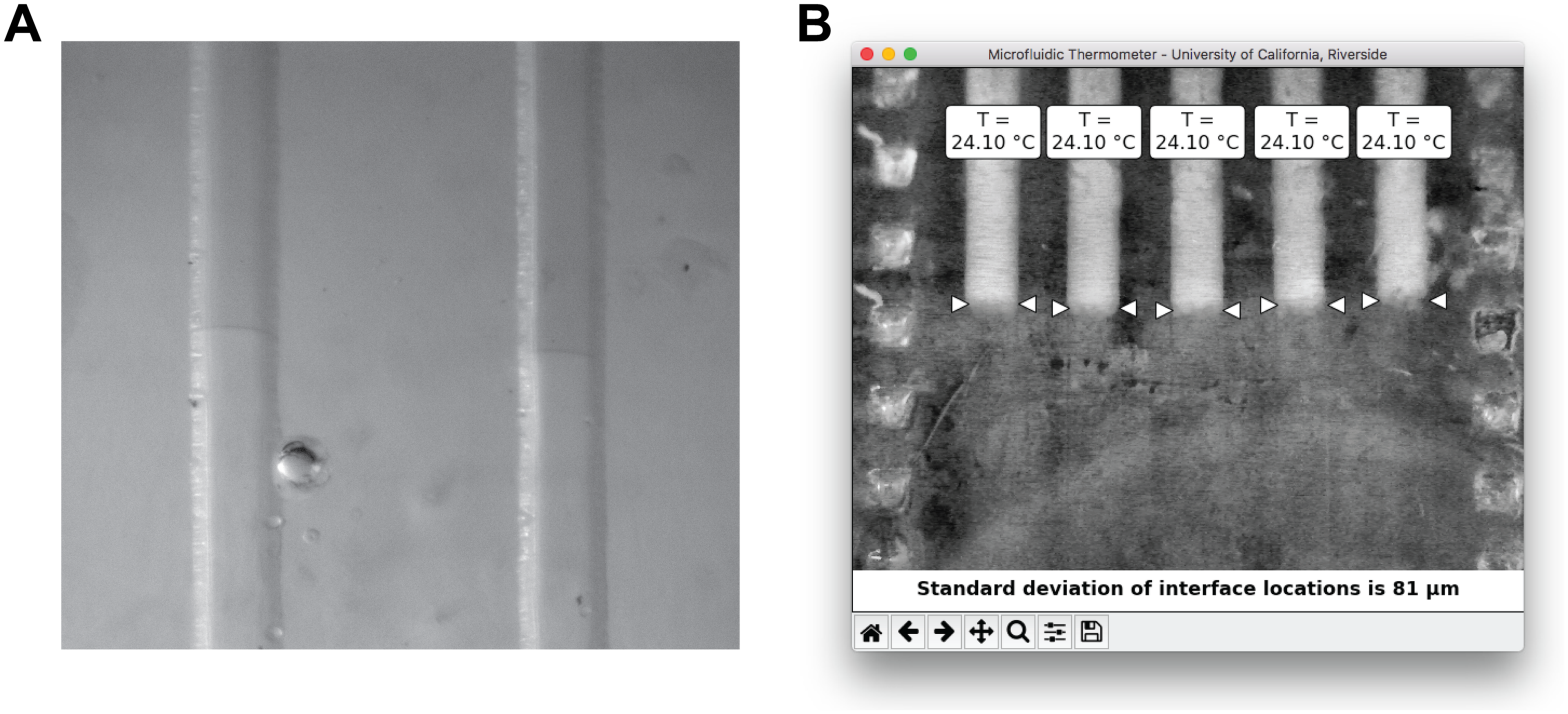
(A) Using a glass microfluidic chip as a microfluidic thermometer. Solid-liquid interfaces in channels containing only 400 nL of water mark the location of the T = 0°C isotherm. (B) Using coconut oil (solidifying/melting point = 24.1°C) in a 3D-pri crofluidic thermometer chip. The interfaces between the solid oil (white) and liquid oil (transparent) are easily identified and form a stable linear isotherm in the chip. Including an additional material with a different freezing/melting point in the thermometer chip would enable measurement of temperatures well above zero Celsius.

We also experimentally verified the linearity of isotherms in microfluidic chips with much smaller channel volumes. Fig 5A shows a closeup of two solid-liquid interfaces in a borosilicate glass chip containing 400 nL of water with a small amount of food coloring in each channel. The solid-liquid interfaces are clearly visible, confirming that microfluidic thermometer chips can be fabricated and used with nanoliter-scale volumes.

To experimentally verify our assumption that the temperature gradient along the chip is linear within the measurement region (and therefore a line along a channel in this region is a thermal isocline), we filled each of the five channels with different fluids with precisely-known freezing/melting points (8%, 6%, 4%, and 2% *m/m* NaCl solutions and deionized water in channels *A*, *B*, *C*, *D*, and *E*, respectively) and established a temperature gradient along the chip. The micrograph of the measurement region in Fig 4B shows that the solid-liquid interfaces appear in different locations along the channels; the material with the lowest freezing point (8% NaCl; channel *A*) has an interface near the bottom of the image, and the material with the highest freezing point (deionized water; channel *E*) has an interface near the middle of the image. After importing this image into our software and clicking on the location of each solid-liquid interface, the software generates a plot of material freezing/melting point vs. vertical location of the solid-liquid interface of the material (inset of Fig 4B). The plot is linear (solid line; *R*^2^ = 0.97) with a maximum difference of 0.48°C between the measured locations of the solid-liquid interfaces and those predicted by a linear regression fit of the measured locations. These results support our assumption that the temperature gradient is linear along the length of the chip within the measurement region. If even higher precision is needed for an application, the solid-liquid interface locations can be fitted to a second-order polynomial (dotted line in Fig 4B inset) which decreases the maximum difference between actual and predicted interface locations to just 0.22°C. However, we used the linear temperature gradient assumption in this work.

### Measuring temperature of an “unknown” solution using the thermometer chip

By utilizing the solid-liquid interface positions of two solutions with known freezing points, we were able to use the microfluidic thermometer chip to measure the temperatures throughout a sample of an “unknown” solution inside the measurement region. Specifically, we used the chip to determine the freezing point of a sodium chloride solution in the chip. Fig 4C shows a photograph of the thermometer chip filled with deionized water in channels *A* and *D* (freezing point *T*_1_ = 0°C), 8.0% (*m/m*) NaCl solution in channels *B* and *E* (freezing point *T*_2_ = −5.08°C), and a solution with a simulated unknown freezing point in channel *C* (4.0% (*m/m*) NaCl solution). After loading the image into our custom software (S2 and S3 Files) and clicking on the locations of each solid-liquid interface, the software uses Eqs 1–6 to determine that the freezing point *T*_3_ of the “unknown” 4% NaCl solution is −2.28 ± 0.26°C (details in S3 File). This value is only 0.13°C higher than the known literature value for the freezing point of a 4% (*m/m*) NaCl solution (−2.41°C [30]).

### Using the microfluidic thermometer at temperatures well above 0°C

The experiments above use water and aqueous sodium chloride solutions to measure temperatures at or below 0°C. However, there are obviously many applications for temperature measurements over a wide range of temperatures, not just below zero Celsius. To demonstrate that the microfluidic thermometer chip is also capable of measuring temperatures well above 0°C, we filled all five channels with coconut oil (which has a precise solidifying/melting point at 24.1°C [31]) and created a stable temperature gradient along the channels as described above. Since the freezing/melting point of coconut oil is slightly above ambient temperature, we heated (not cooled) one end of the microfluidic thermometer chip by reversing the polarity on the thermoelectric cooler and did not need dry nitrogen to prevent condensation.

Fig 5B shows the results from using our custom software (S2 and S3 Files) to analyze the linearity of the isotherm in the microfluidic thermometer chip while filled with coconut oil. The interfaces between the solid oil (white) and liquid oil (transparent) are very easy to locate, and the standard deviation of the locations of these interfaces along the channels, 81 *μ*m, is actually lower than that observed using water in Fig 4A. This shows that the solid-liquid interfaces in coconut oil form stable linear isotherms in the thermometer chip. By also using *e.g*. a second oil with a slightly different freezing/melting point, one can use the microfluidic thermometer chip to measure temperatures well above zero Celsius.

## Conclusions

In this work, we demonstrated a simple technique for making precise measurements of the temperatures of microliter- and nanoliter-scale volumes. This technique requires minimal equipment and no probes, labels, or other modifications to the sample being measured. We used this technique to measure the freezing point of a simulated unknown solution. By using materials with different known freezing/melting points, our technique can be tailored for measurements at many different temperatures. Additionally, by using 3D printing to fabricate our thermometer chip, any researcher can download the design of the chip (S1 File) and fabricate and use the chip. As the glass microfluidic thermometer chip in Fig 5 shows, this method is not limited to 3D-printed microfluidic devices and is suitable for use in any fabrication method that provides optical access to the channel contents. Finally, since the freezing point of a substance is an intrinsic property of that substance, our technique could be used as a simple way to identify a substance (or rule out other substances) by accurately measuring its freezing point.

In its current form, the thermometer chip is limited to making temperature measurements within the measurement region (the area of the chip where the temperature gradient is linear; dotted boxes in Fig 2A and 2B and gray region in Fig 2E). This roughly 6-mm-long region contains a linear temperature gradient that spans about 5 degrees Celsius. The *location* of this temperature range on the temperature scale can be set at will by loading the microfluidic thermometer channels with materials with different freezing/melting points (for example, using aqueous sodium chloride solutions to measure temperatures around 0°C as in Figs 4 and 5A, or using coconut oil to measure temperatures around 24°C as in Fig 5B). However, we cannot use sodium chloride solutions *and* coconut oil *simultaneously* in the thermometer chip because the freezing/melting points of these substances differ by over 24°C—that means that at least one of the substances’ solid-liquid interfaces would lie far outside the measurement region. If one of the solid-liquid interfaces forms outside of the region where the temperature gradient is linear, we would not be able to accurately predict the temperatures *between* the different solid-liquid interfaces. Therefore, our technique is more suited for precisely measuring temperatures in a narrow range using similar materials, not measuring temperatures in a wide range using dissimilar materials.

## Supporting information

S1 File Design of the microfluidic thermometer chip in the standard.STL format used by most 3D printers. (STL)

S2 File microfluidic_thermometer.py, a custom Python program that automates the data analysis required when using the microfluidic thermometer chip. Used to generate Figs 1D and 1E, 4 and 5B.
(ZIP)

S3 File User guide for microfluidic_thermometer.py. Step-by-step description of using microfluidic_thermometer.py to perform the analyses in Fig 4.
(PDF)
