# Supplementary figures and images for: A microfluidic thermometer: Precise temperature measurements in microliter- and nanoliter-scale volumes
Source: PLoS One. 2017 Dec 28;12(12):e0189430. doi: 10.1371/journal.pone.0189430 (PMC5746210; doi:10.1371/journal.pone.0189430)

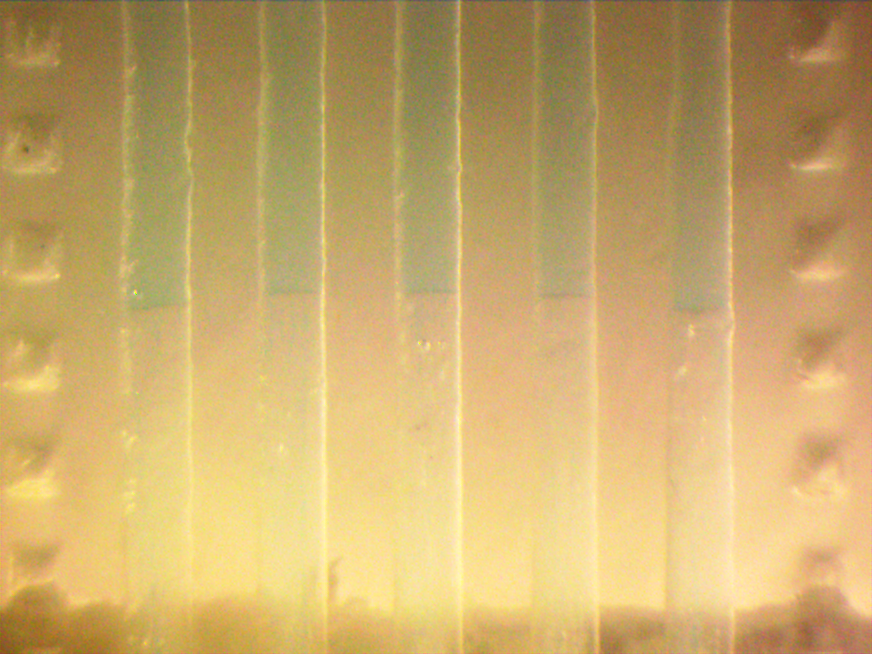

Supplement: S2 File — Used to generate Figs 1D and 1E, 4 and 5B. (ZIP) [file pone.0189430.s002.zip › 00000.png]

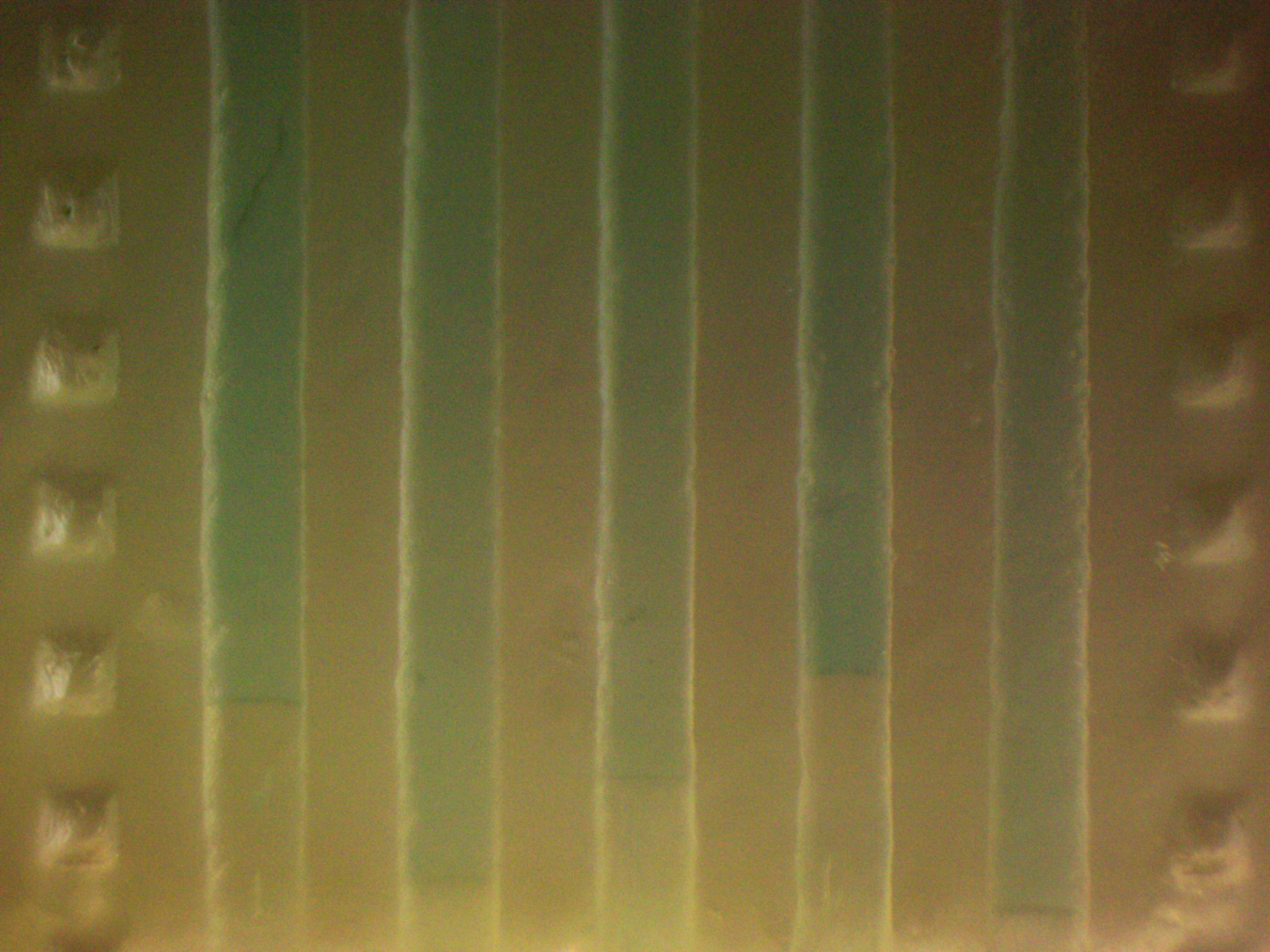

Supplement: S2 File — Used to generate Figs 1D and 1E, 4 and 5B. (ZIP) [file pone.0189430.s002.zip › 08408.png]

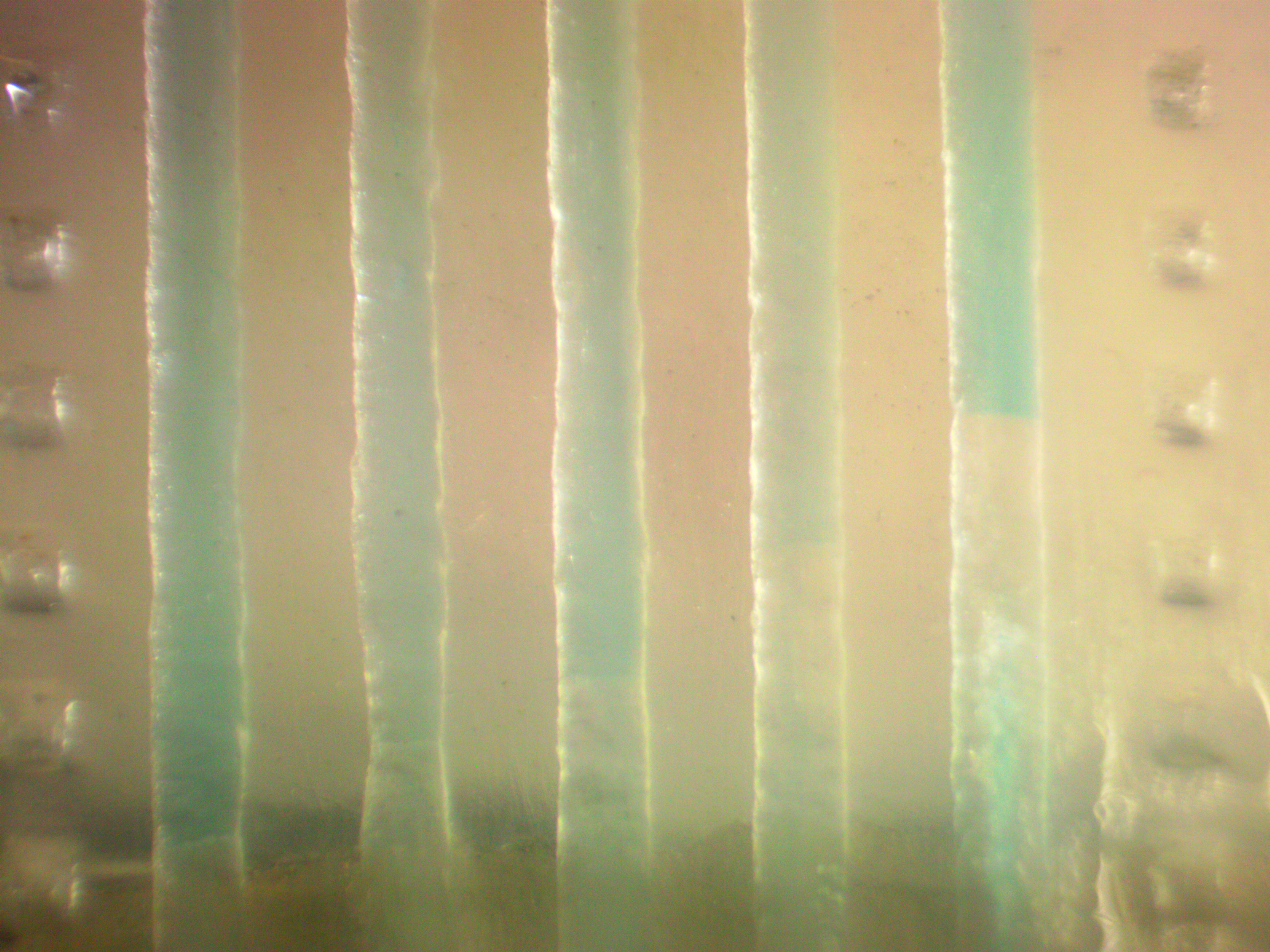

Supplement: S2 File — Used to generate Figs 1D and 1E, 4 and 5B. (ZIP) [file pone.0189430.s002.zip › 86420.png]

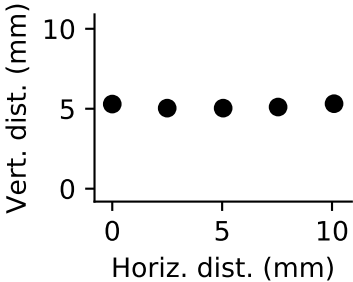

Supplement: S2 File — Used to generate Figs 1D and 1E, 4 and 5B. (ZIP) [file pone.0189430.s002.zip › fig_4a_1.pdf]

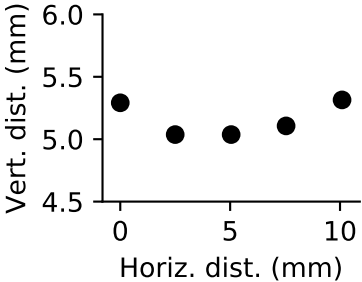

Supplement: S2 File — Used to generate Figs 1D and 1E, 4 and 5B. (ZIP) [file pone.0189430.s002.zip › fig_4a_2.pdf]

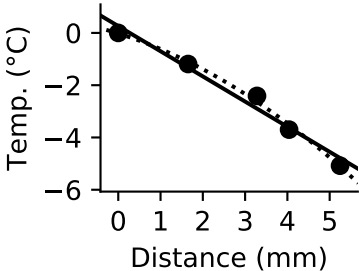

Supplement: S2 File — Used to generate Figs 1D and 1E, 4 and 5B. (ZIP) [file pone.0189430.s002.zip › fig_4b.pdf]
