# Supplementary material for: A microfluidic thermometer: Precise temperature measurements in microliter- and nanoliter-scale volumes
Source: PLoS One. 2017 Dec 28;12(12):e0189430. doi: 10.1371/journal.pone.0189430 (PMC5746210; doi:10.1371/journal.pone.0189430)
Supplement: S3 File — Step-by-step description of using microfluidic_thermometer.py to perform the analyses in Fig 4. (PDF) [file pone.0189430.s003.pdf]

Using `microfluidic_thermometer.py`

Supplementary information for "A microfluidic thermometer: Precise temperature measurements in microliter- and nanoliter-scale volumes"

by Brittney McKenzie and William H. Grover,
Department of Bioengineering, University of California, Riverside, CA

A custom Python program, `microfluidic_thermometer.py`, was written to analyze images of the microfluidic thermometer chip. The current version of the software is available as online Supplementary Information, and the latest version of the software is available for download from `http://groverlab.org`. Sample images of the microfluidic thermometer chip are included for use with the software. To use the software to measure the unknown freezing point of a solution, the user creates a short Python script describing the images to be analyzed. For example, to create Figure 4 in the main text, the following script was used:

```
import microfluidic_thermometer as mt

filename = "00000.png"
mt.get_scale(filename, distance=2.5)
mt.calibrate_one_concentration(filename,
                               freezing_point_1=0)

filename = "86420.png"
mt.get_scale(filename, distance=2.5)
mt.calibrate_five_concentrations(filename,
                                 freezing_point_1=-5.08,
                                 freezing_point_2=-3.70,
                                 freezing_point_3=-2.41,
                                 freezing_point_4=-1.19,
                                 freezing_point_5=0.0)

filename = "08408.png"
mt.get_scale(filename, distance=2.5)
mt.measure_unknown(filename,
                   freezing_point_1=0,
                   freezing_point_2=-5.08)

mt.report("report.txt")
```

Line 1 imports `microfluidic_thermometer.py` (which should be in the same directory as this script).

Line 3 specifies the name of the first image file used in this analysis. In this case, the file `00000.png` is a photograph of the microfluidic thermometer chip with all five channels filled with water:

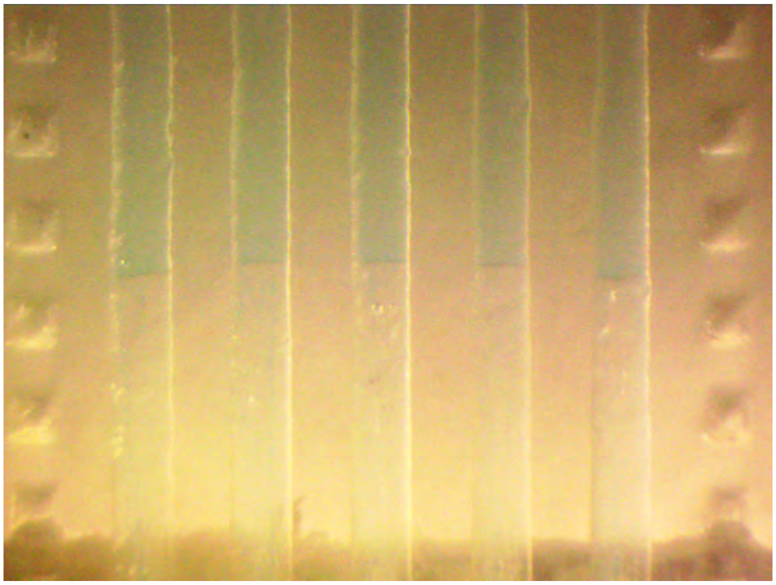

Line 4 calls the `get_scale` function, which is used to determine the scale of the image. This is necessary if subsequent images were acquired at a different magnification or pixel resolution than the current image. The argument `distance = 2.5` tells the code the actual length of a known feature on the chip; in this case the distance between two channel edges in the microfluidic thermometer is 2.5 millimeters. When this script is executed, Line 4 makes a window appear:

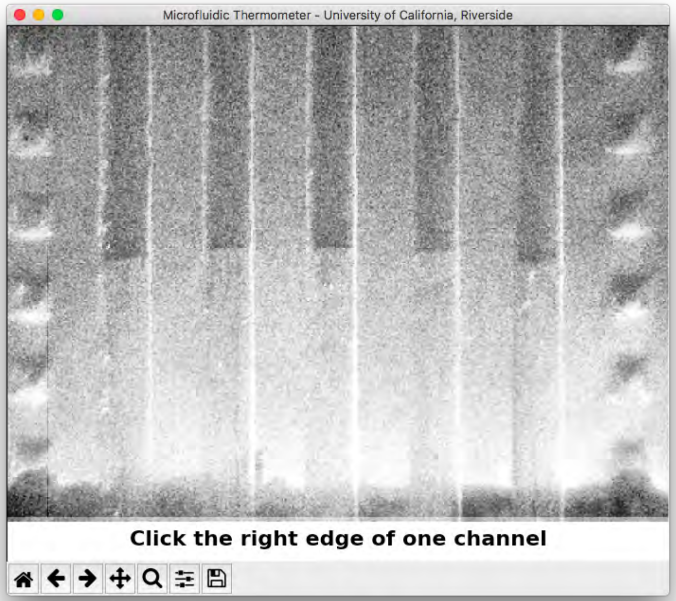

The window instructs the user to “click on the right edge of one channel.” After the edge is clicked, a triangular marker is placed on the edge and the user is instructed to “click the right edge of an adjacent channel:

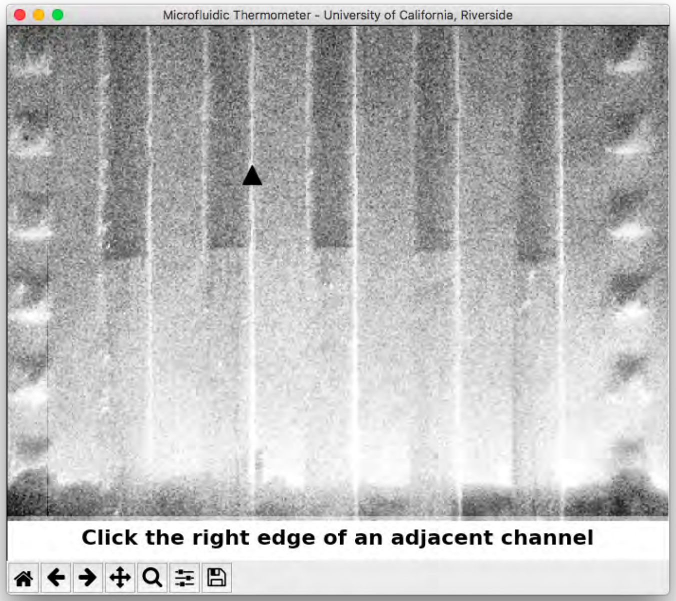

After the second edge is clicked, another triangular marker is placed on the edge, and the

scale of the image is reported to the user ("Image scale is 19 μm/pixel") and is saved for future use:

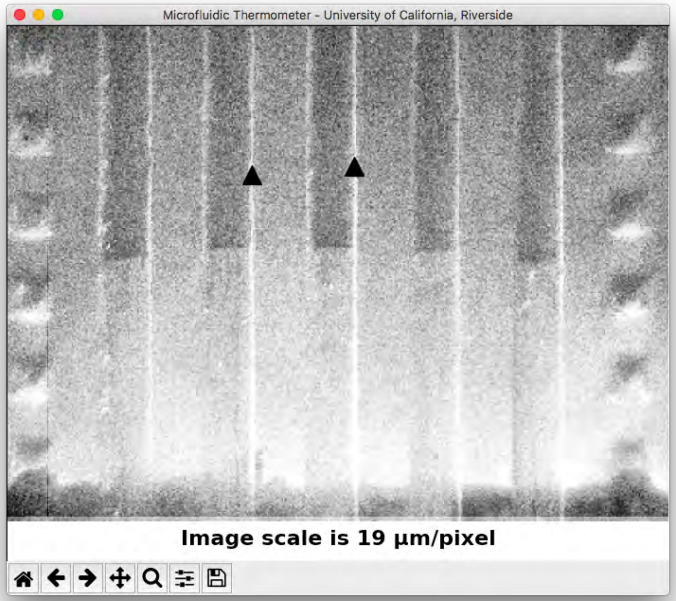

Line 5 calls the `calibrate_one_concentration` function, which is used to analyze the uniformity of an isotherm across the channels in the thermometer chip. The argument `freezing_point_1 = 0.0` tells the code the known freezing point of the solution in all five channels of the chip; in this case the channels are filled with water so the freezing point is 0°C. The window instructs the user to "click the first interface with freezing point 0°C":

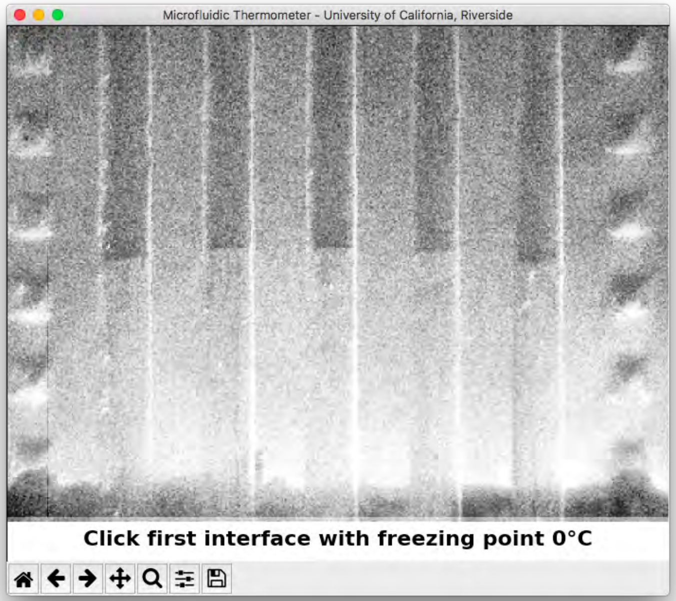

After the user clicks on the solid-liquid interface in the first channel, triangular markers and a label are added to the plot to indicate the known temperature at the interface, and the user is instructed to "click second interface with freezing point 0°C":

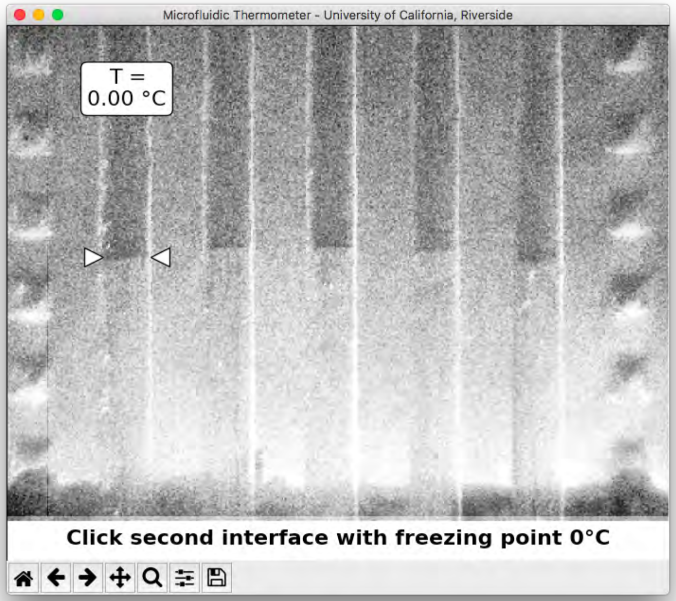

This process repeats, instructing the user to click the third solid-liquid interface:

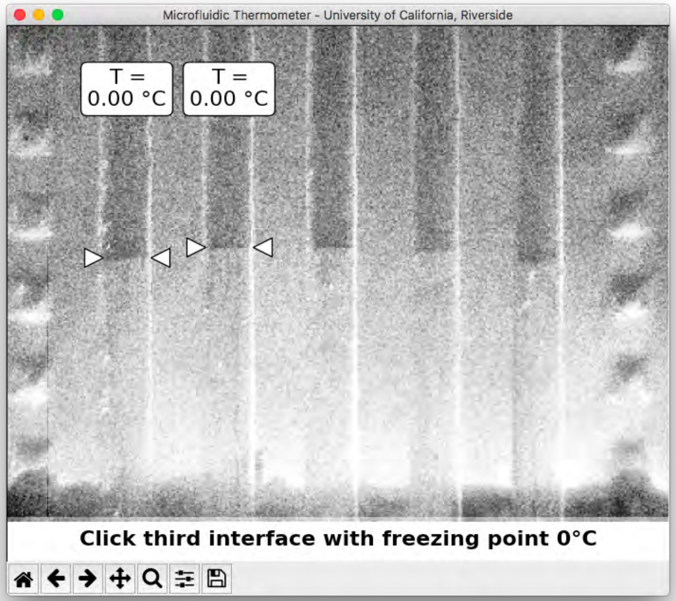

the fourth interface:

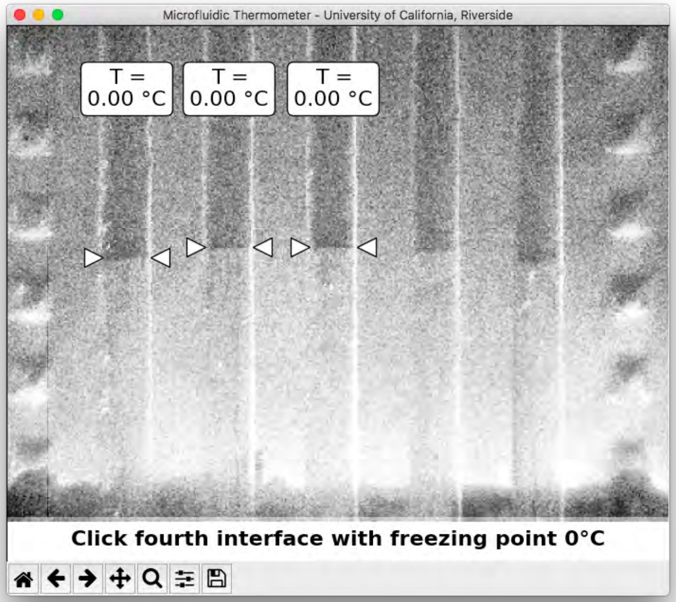

and the fifth interface:

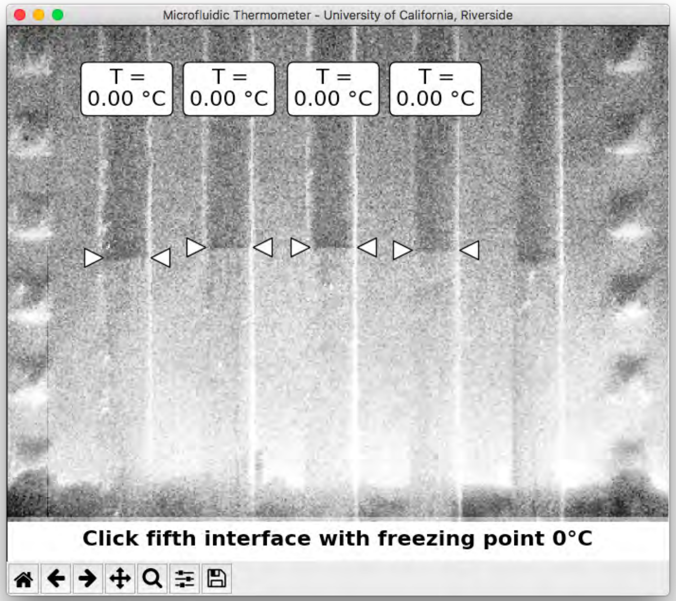

The variation of the interface locations in the vertical dimension is reported to the user ("Standard deviation of the interface locations is 122 μm") and is saved for future use:

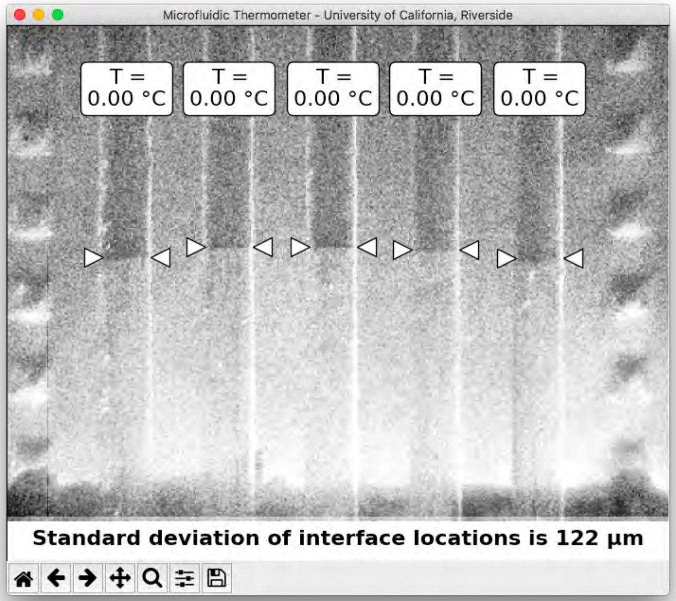

Finally, the software automatically saves two plots (normal and closeup) of the vertical location of each solid-liquid interface vs. the horizontal location of the interface:

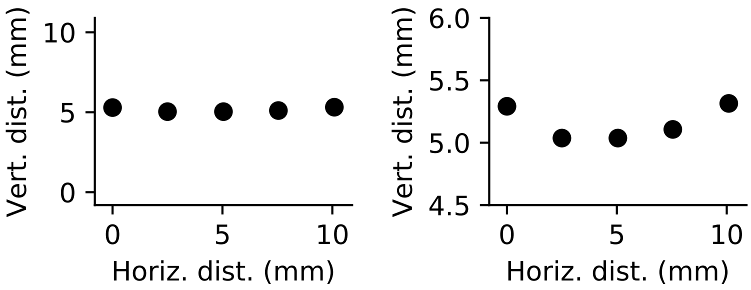

These plots confirm that isotherms are roughly linear in the measurement region of the thermometer chip, with a variation in vertical interface location of less than 500 μm over a 10 mm wide region.

Line 8 specifies a new image file to be used. File `86420.png` is a photograph of the microfluidic thermometer with an 8% *w/w* solution of sodium chloride in channel A, a 6% solution in channel B, a 4% solution in channel C, a 2% solution in channel D, and a 0% solution (water) in channel E:

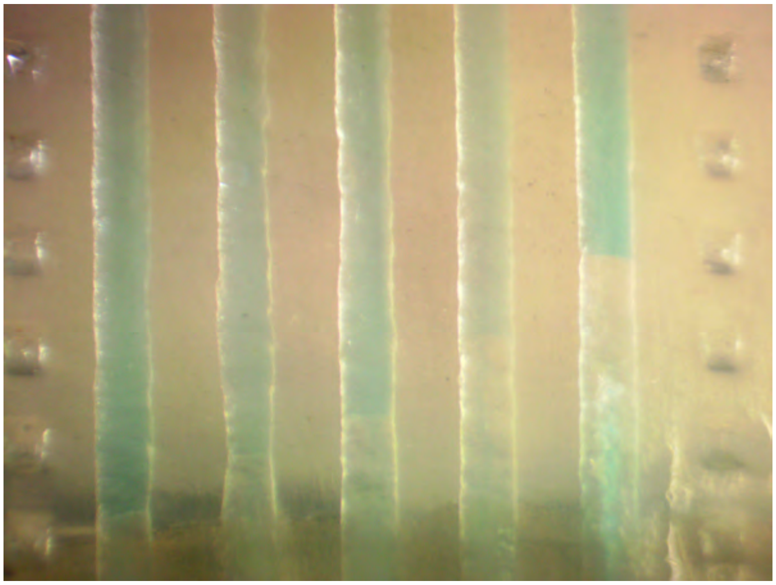

Line 9 again calls the `get_scale` function to determine the scale of the new image. The user clicks on two channel edges, and the scale is reported to the user and saved for future use:

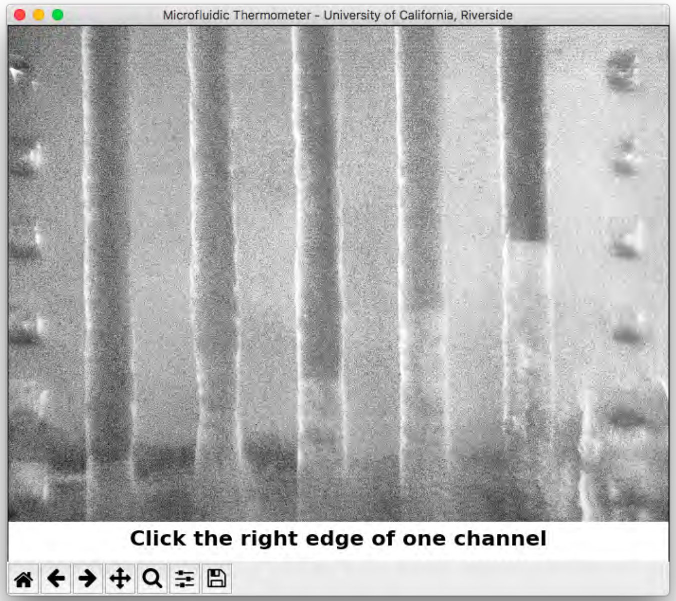
Microfluidic Thermometer - University of California, Riverside
Click the right edge of one channel

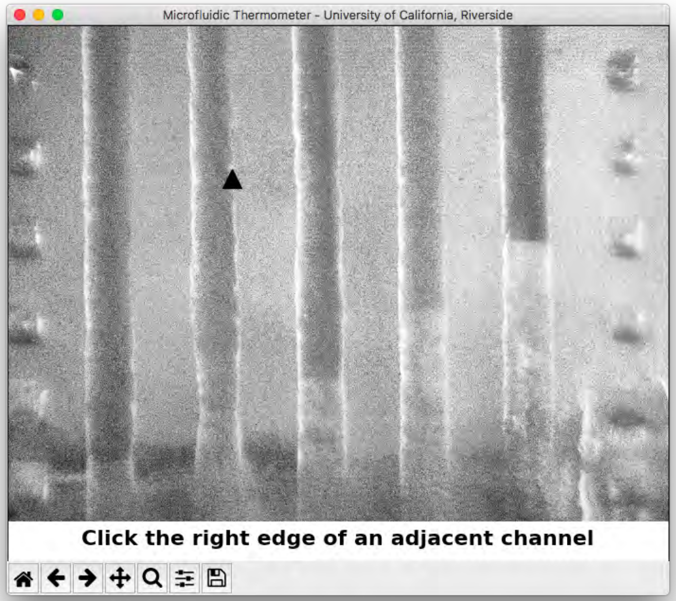
Microfluidic Thermometer - University of California, Riverside
Click the right edge of an adjacent channel

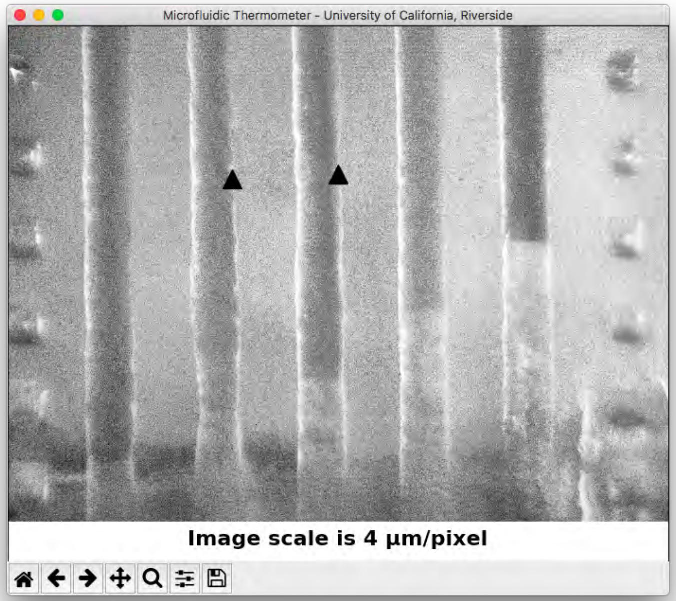

Line 10 calls the `calibrate_five_concentrations` function, which is used to analyze the shape of the temperature gradient along the channels in the thermometer chip. The arguments `freezing_point_1 = -5.08`, `freezing_point_2 = -3.70`, `freezing_point_3 = -2.41`, `freezing_point_4 = -1.19`, and `freezing_point_5 = 0.0` specify the freezing points of the five sodium chloride solutions in channels A through E. The window then instructs the user to click on each of the five solid-liquid interfaces in turn:

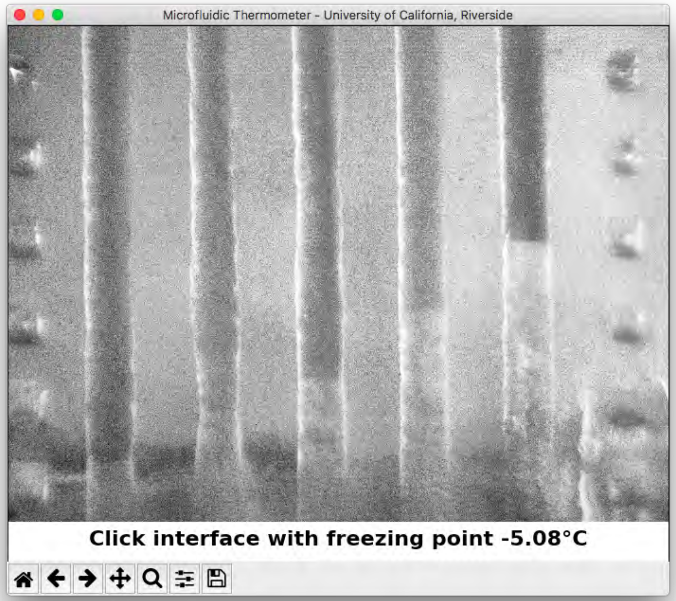
Microfluidic Thermometer - University of California, Riverside
Click interface with freezing point -5.08°C

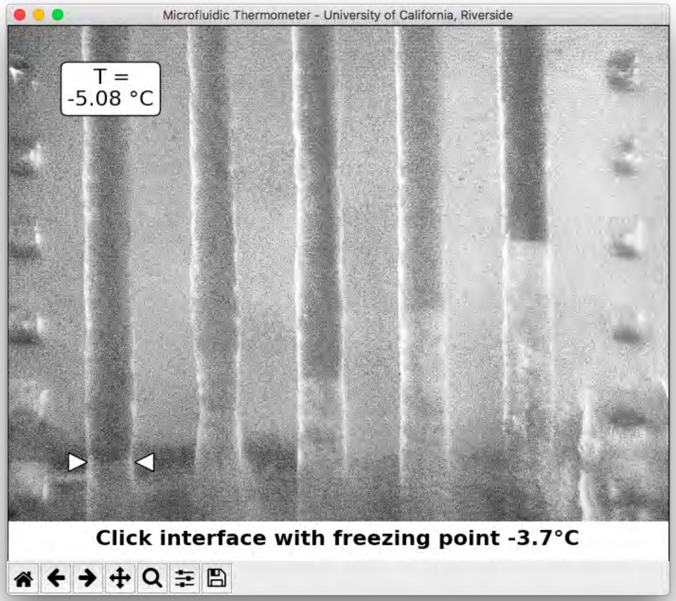
Microfluidic Thermometer - University of California, Riverside
T =
-5.08 °C
Click interface with freezing point -3.7°C

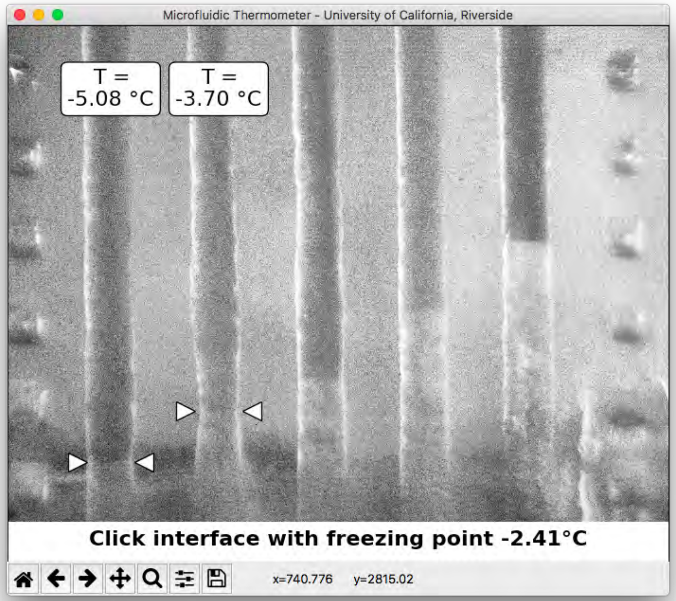
Microfluidic Thermometer - University of California, Riverside
T = -5.08 °C
T = -3.70 °C
Click interface with freezing point -2.41°C
x=740.776 y=2815.02

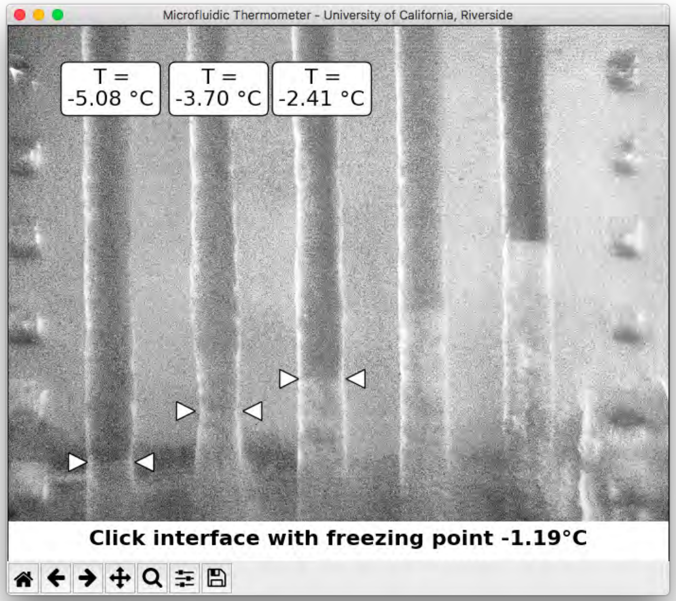
Microfluidic Thermometer - University of California, Riverside
T = -5.08 °C
T = -3.70 °C
T = -2.41 °C
Click interface with freezing point -1.19°C

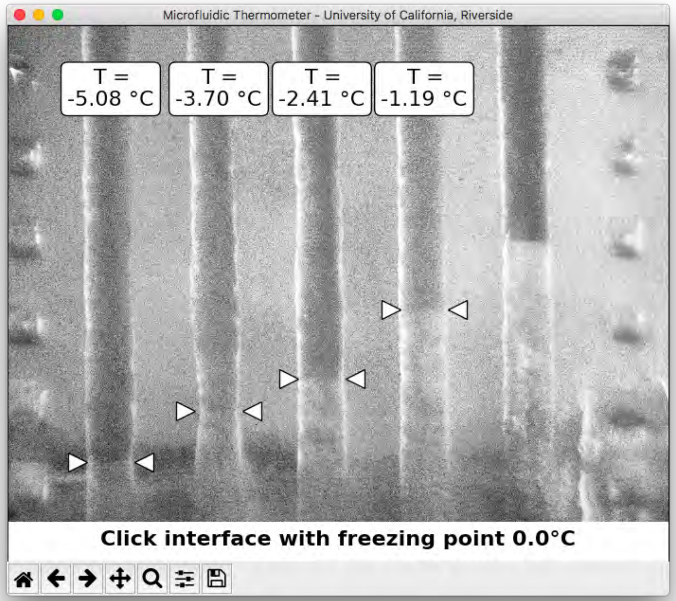

The R^2 value of the linear fit of a plot of interface location vs. temperature is reported to the user ("R-squared = 0.97"):

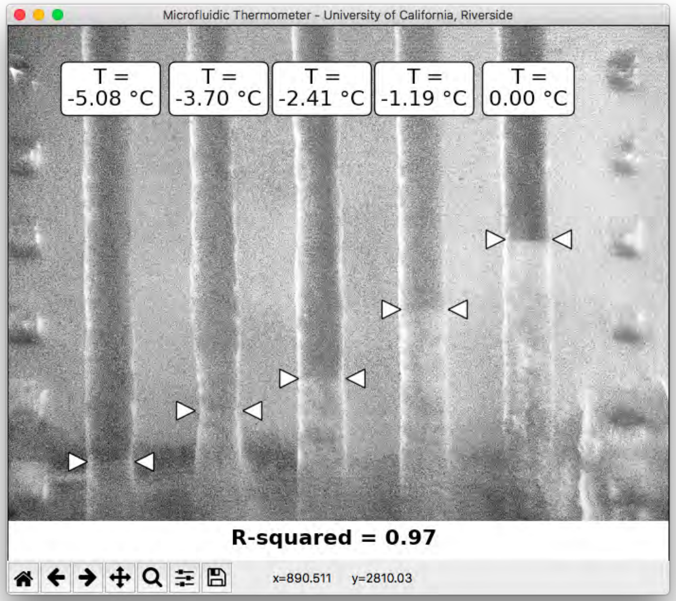

Finally, the software automatically saves a plot of solid-liquid interface temperature vs. the location of that interface in the vertical direction on the chip:

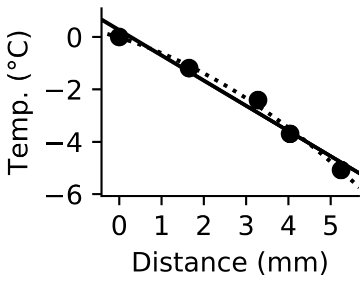

The plot includes two least-squares regression fits, one linear (the solid line, which deviates from the measured temperature values by less than 0.48 °C) and one second-order polynomial (the dotted line, which deviates from the measured temperature values by less than 0.22 °C). This plot confirms that the temperature gradient is roughly linear in the measurement region of the thermometer chip, and other functions (like the second-order polynomial) can be used if additional accuracy is needed).

Line 17 specifies a new image file to be used in the rest of the analysis. In this case, the file `08408.png` is a photograph of the microfluidic thermometer chip with water in channels A and D, an 8% *w*/*w* solution of sodium chloride in channels B and E, and an solution with "unknown" freezing point in channel C:

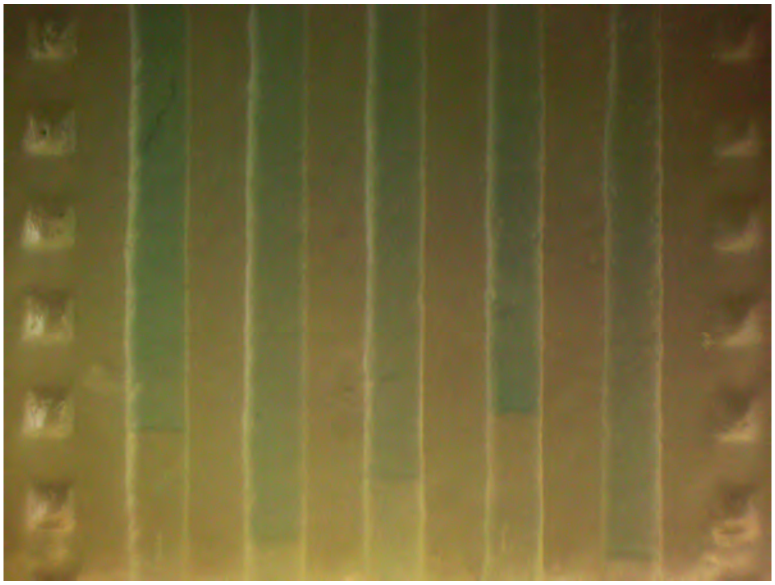

Line 18 again calls the `get_scale` function to determine the scale of the new image. The user clicks on two channel edges, and the scale is reported to the user and saved for future use:

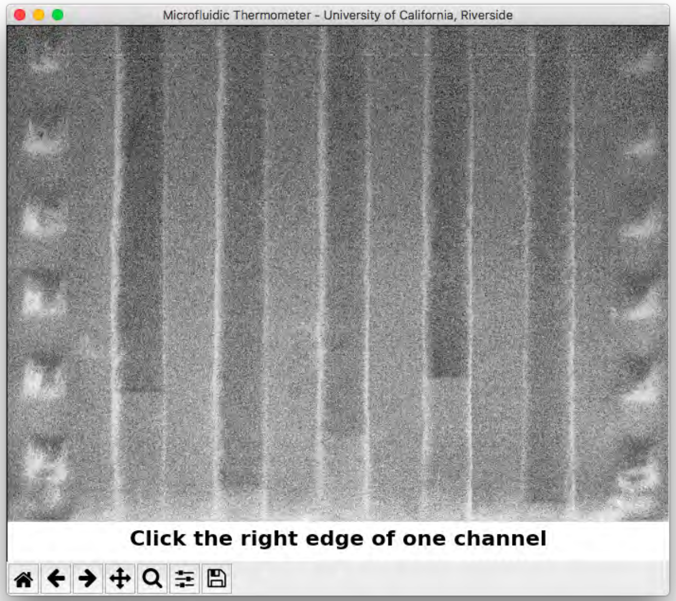
Microfluidic Thermometer - University of California, Riverside
Click the right edge of one channel

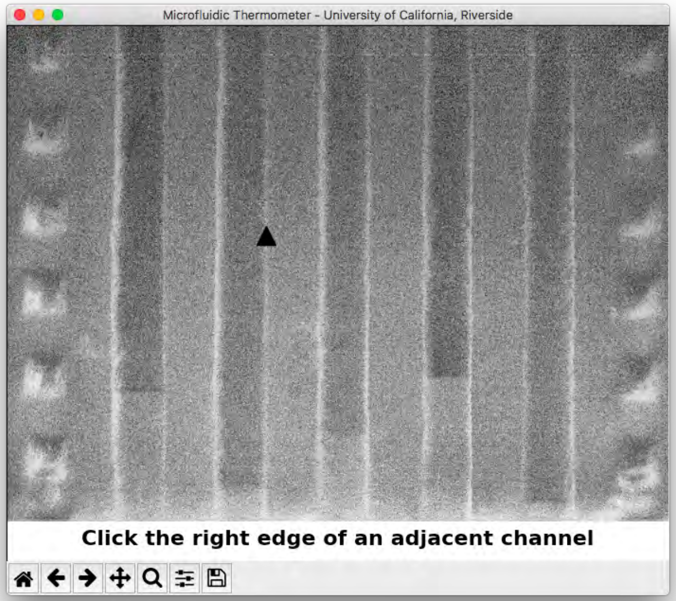
Microfluidic Thermometer - University of California, Riverside
Click the right edge of an adjacent channel

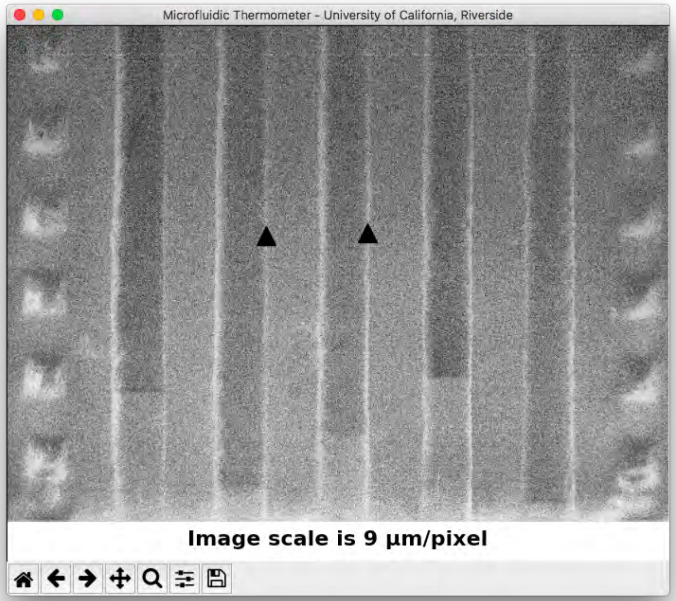

Line 19 calls the `measure_unknown` function, which is used to measure the freezing point of a solution when the other four channels are filled with two solutions with known freezing points. The arguments `freezing_point_1 = 0.0` and `freezing_point_2 = -5.08` tell the code the known freezing point of the solution in channels A and D is 0°C and the known freezing point of the solution in channels B and E is −5.08°C. The window instructs the user to click the locations of each solid-liquid interface in the chip, first the interfaces at freezing point 0°C:

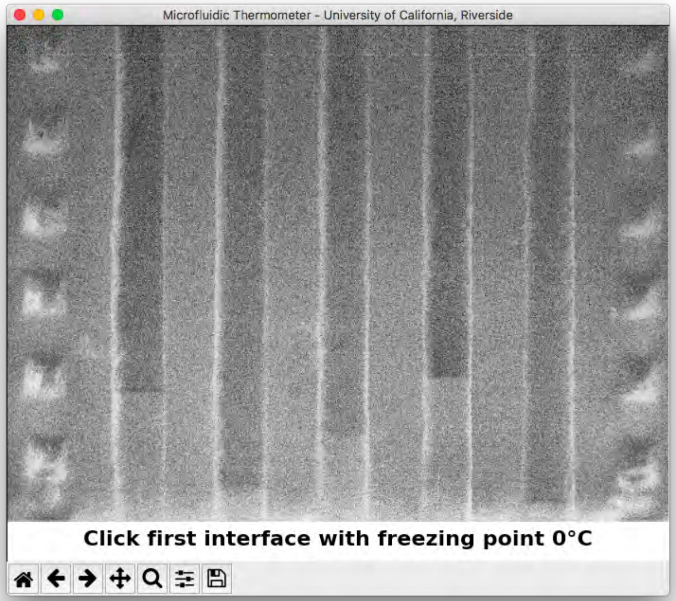

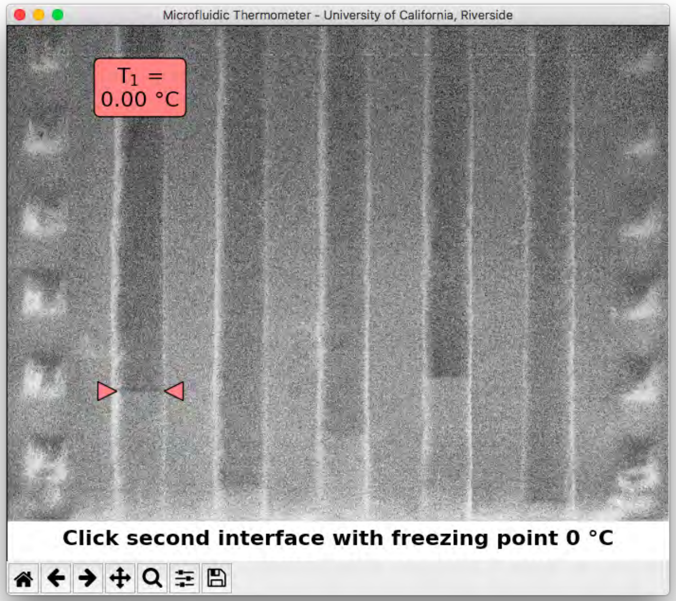

then the interfaces at freezing point -5.08°C:

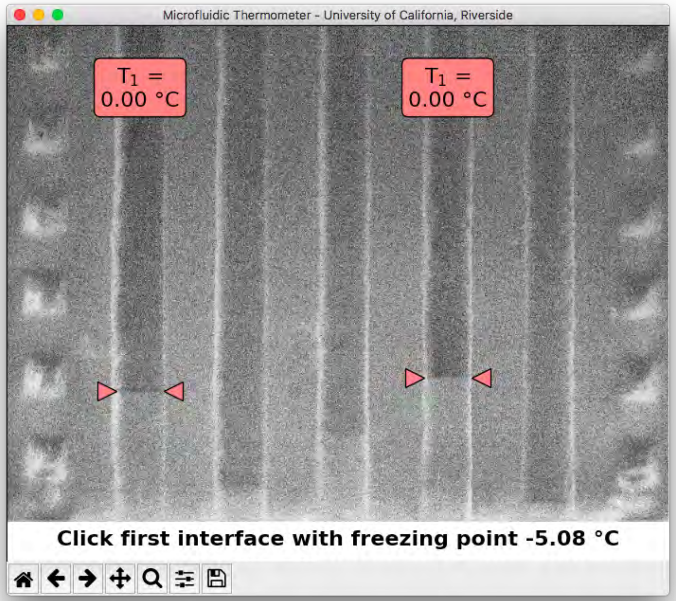

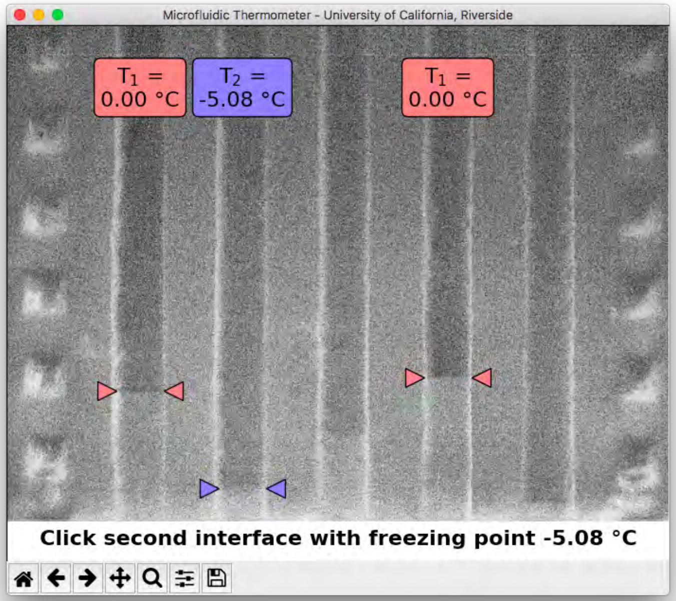

and finally the interface in the channel containing the "unknown" freezing point solution:

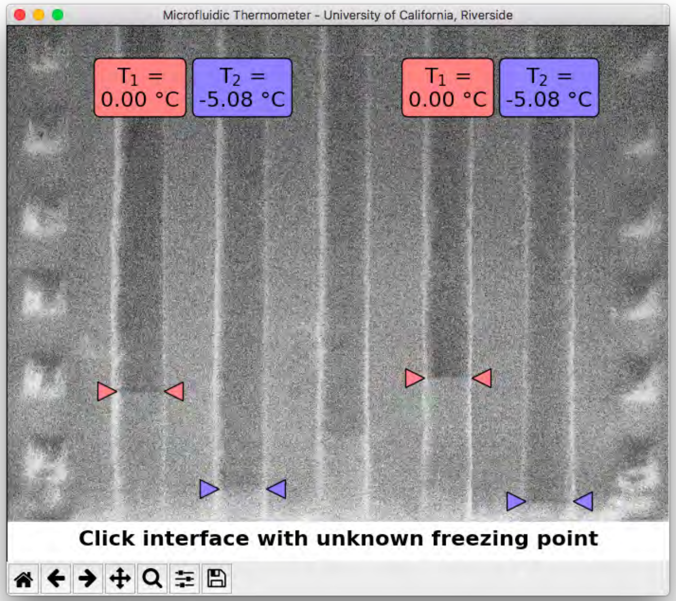

Finally, the freezing point of the "unknown" solution is reported to the user ("freezing point of unknown is $-2.28°C$"):

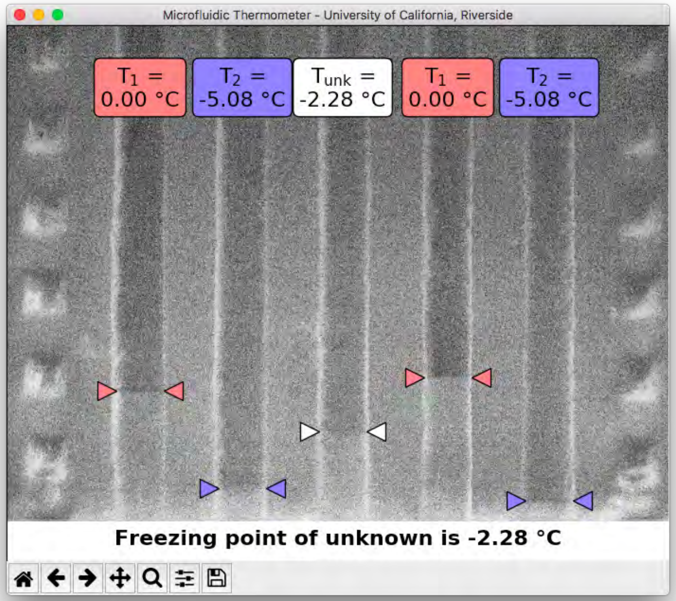

Line 23 uses the `report` function to save a summary of the analysis of these three images in a text file with the specified name.
